# Clinical Examination, Diagnosis, and Conservative Treatment of Chronic Low Back Pain: A Narrative Review

**DOI:** 10.3390/life14091090

**Published:** 2024-08-29

**Authors:** Paulien Custers, Erik Van de Kelft, Bart Eeckhaut, Wouter Sabbe, An Hofman, Annick Debuysscher, Gilles Van Acker, Gaethan Maes

**Affiliations:** 1 Department of Physical Medicine and Rehabilitation, Ghent University Hospital, Corneel Heymanslaan 10, 9000 Ghent, Belgium; 2 Department of Physical Medicine and Rehabilitation, VITAZ, Moerlandstraat 1, 9100 Sint-Niklaas, Belgium; 3 Department of Neurosurgery, VITAZ, Moerlandstraat 1, 9100 Sint-Niklaas, Belgium

**Keywords:** chronic non-specific low back pain, low back pain, CNSLBP, conservative treatment

## Abstract

Chronic low back pain is one of the most frequent reasons for medical consultation. It is important to make the correct diagnosis to select the most appropriate treatment in a stepwise approach. In this narrative review, we focus on the clinical examination, the diagnosis, and the conservative treatment of chronic non-specific low back pain. Belgian guidelines for low back pain were used as a basis, followed by a snowball search starting from two articles. Besides that, the Cochrane database was consulted using the following research areas: “multidisciplinary biopsychosocial rehabilitation”, “physical examination of lumbar spine”, and “rehabilitation back pain”. Lastly, we took information from three handbooks. The diagnosis of low back pain starts with a thorough history, including red, yellow, orange, black, and blue flags. Physical, neurological, sensory, and motor testing is performed and complemented with specific tests for low back pain. With a focus on the conservative treatment, pharmacological and non-pharmacological treatments are possible. For CNSLBP, conservative management is advised, starting with reassurance and clear patient education about the condition. While additional treatments, such as manipulation, massage, and acupuncture, can be considered, their effectiveness is not well supported by evidence. Our center emphasizes exercise within a multidisciplinary biopsychosocial rehabilitation program, and although evidence for this approach is limited, we have seen positive outcomes, including improved mobility, strength, and higher return-to-work rates, particularly with the David Spine Concept (DSC).

## 1. Introduction

Low back pain significantly impacts patients’ quality of life. Research by Sciensano in 2018 revealed that 2.3 million healthy life years were lost in Belgium due to 37 diseases, with low back pain being one of the top 5 contributors to this loss (6.2%) due to its significant reduction on quality of life (13.2%) [1]. Low back pain has a lifetime prevalence as high as 84%, occurring most frequently between the ages of 50 and 55, and is more prevalent in women. Chronic low back pain has an incidence of 10–15% in the population, making it one of the leading causes of work loss and a significant economic burden, thus constituting a global public health issue [2,3,4,5]. This highlights the importance of accurate diagnosis and a comprehensive overview of potential treatment options.

It is crucial to distinguish between specific and non-specific low back pain. Specific low back pain, which accounts for only 10% of patients with CLBP, has a well-known pain mechanism. The pain can be caused by infection, trauma, tumor, or inflammation. In such cases, a ‘red flag’ is added to the patient’s history of CLBP [2,6,7,8,9]. If there is also irradiating pain in the legs, the origin of this pain must be clarified. First, the difference between radicular pain and radiculopathy must be clarified. Radicular pain refers solely to pain radiating in a dermatomal distribution, while radiculopathy involves objective sensory, motor, and/or reflex loss [9,10]. In addition, radiating pain in the legs can originate from the facet joints, the sacroiliac joint, piriformis syndrome, hip pathology, or polyneuropathy. In non-specific low back pain, the pain mechanism is unclear [4,6,9]. 

This narrative review provides an overview of the approach to chronic non-specific low back pain (CNSLBP) from the perspective of physical medicine and rehabilitation. The history, clinical examination, and conservative treatment options will be discussed. The objective is to provide a comprehensive overview of the current evidence, if any.

## 2. Method

In this narrative review, the following method was used. First of all, the Belgian guidelines for low back pain were used [7]. Secondly, we used the snowball search method, starting from two articles [10,11]. Thirdly, the Cochrane Library was used with the following search areas: “low back pain”, “multidisciplinary biopsychosocial rehabilitation”, “physical examination of lumbar spine”, and “rehabilitation back pain”. Lastly, we took information from three handbooks: *Clinical Sports Medicine*, *Braddom’s Physical Rehabilitation and Medicine* (two editions), and *Surgery of the Spine and Spinal Cord* [2,12,13,14]. There were no restrictions on publication years, and all articles reviewed were written in English.

## 3. Diagnosis

It is important to start with a thorough history and clinical examination [2,9]. These are the leading factors for performing extra investigations and for setting up the treatment protocol. It is important to know that the value of the history and clinical examination is confounded by the absence of a golden standard.

### 3.1. History 

When starting the history of the patient’s back pain, it is favorable to use a standardized approach. The following points have to be checked [2,5,15]:

When did the pain start? What is the duration of the pain? 

Where is the pain located? 

What is the character/quality of the pain (i.e., aching, hot/burning, sharp, throbbing, shooting, stabbing, and tiring/exhausting)?

Are there aggravating and alleviating factors? 

Is there any irradiation? 

When does the pain occur? Is the pain always there? 

What is the severity of the pain? (i.e., NRS scale/VAS scale can be used [2,7])

Do you have pain at night?

Are there associated signs and symptoms?

Which treatments have you tried up till now?

For surgeons, it is important to identify mechanical LBP—pain disappears in a specific position, mostly when lying down. If the patient cannot find a position in which the pain almost disappears, the surgeon cannot either. By interpreting the timing of back pain, a distinction must be made between acute (<6 weeks), subacute (>6 weeks but <3 months), and chronic low back pain (>3 months) [13].

Importantly, the “red flags” must be excluded. Red flags are elements of the history that are suggestive of an underlying serious condition. They have to be interpreted in the clinical context of the patient’s history to not miss a severe spinal pathology, such as cancer, infection, tumor, significant neurologic injury, or fracture [2,6,7,8,9,13,15]. An overview of these “red flags” is presented in the following table in order of urgency (Table 1) [7]. 

Ten percent of acute low back pain becomes chronic. That is why it is important to identify the patients with a high risk of chronic pain [2,5,7]. To have an idea of the risk of chronicity, the psychological, psychiatric, contextual, and work-related factors must be considered. The Belgian guidelines mention yellow, orange, black, and blue flags. Yellow flags are the indicators that include beliefs and perceptions about pain, as well as emotional responses to it [2,6,7,8,13,16]. Secondly, orange flags refer to psychiatric symptoms, such as depression or personality disorders. Thirdly, black flags involve systemic or contextual factors, such as professional or family issues. Lastly, blue flags relate to how individuals perceive the relationship between their work and health [7]. See Table 2 for a summary of the yellow, orange, black, and blue flags. Using these will provide insight into what patients believe is causing the pain, the fear that comes with it, the expectations about the pain and the treatment, or how the pain affects their lives [2,5].

The Tampa scale is used for kinesiophobia, which provides an impression of the degree of pain-related fear in patients with low back pain. To gain an idea of the presence of orange flags, the Zung Self-Rating Depression Scale is used. This scale has the purpose of measuring the degree of depression. 

CNSLBP can have an important influence on the functional status and activity of daily living. In our center, we use the Oswestry Low Back Pain Disability Questionnaire (OLBPDQ) to gain an idea of the functional impairment experienced by the patient due to back pain. The questionnaire consists of the following subjects: pain, self-care (washing, dressing, etc.), lifting, walking, sitting, standing, sleeping, sex life, social life, and travel/transport. 

### 3.2. Physical Examination

In this section, we discuss the protocol that is used at our hospital for the physical examination of lumbar pathology and compare it with the literature. It is important to also examine the hips and sacroiliac joint, to include them in the differential diagnosis.

#### 3.2.1. Observation

The observation begins the moment the patient is called into the consultation room. The physician looks at how the patient stands up from his or her seat and walks in. The next thing to do is to ask the patient to fully undress, except for the underwear. We observe the muscle mass, survey the skin, the bony structures, and the position of the lumbar spine [2,5,15]. Sagittal and coronal balance, hip retroversion, hip extension, knee flexion, pelvic obliquity, and the progression of scoliosis during forward flexion must be thoroughly assessed. Any leg length discrepancy should also be included. 

#### 3.2.2. Palpation

When performing the superficial and deep palpation, the aim is to determine the level of pathology. We palpate the spinous processes of the lumbar spine, the facet joints, sacroiliac joints, the articular joints of the hips, and the trochanter region. Here, we want the patient to tell us when they experience (recognizable) pain. 

The first question that should be asked here is: can a physician accurately find the specific level of the lumbar spine? In a study by Nolet et al., the specific level of the spinous process could be identified in 47% of the cases when using radiographic confirmation [17]. It is important to note that there are no studies on the validity of the tests, so the value of palpation must be questioned [18]. 

Nevertheless, palpation is a standard element of the physical examination in clinical practice to try and determine the painful lumbar level (discal or facet) [2,13,14,15]. 

#### 3.2.3. Range of Motion 

When examining the range of motion, the active and passive flexion, extension, rotation, and lateral bending of the lumbar spine are observed. While doing this, not only the range of motion but also the pain is questioned. The range of motion can be measured with a single or double inclinometer, but mostly it is performed in a semi-quantitative fashion. Pain while performing a flexion maneuver, or when going from flexion to the neutral position (Gower sign), is often related to discal pathology, and pain with extension is associated with posterior pathology or spinal canal stenosis [2]. It is important to note that flexion is a complex movement in which the hamstring muscles and sacroiliac joints can also limit the range of motion [19]. There are no standard values for range of motion available in the literature to compare with, but side-to-side comparison is relevant. 

The Schober index can also be measured, which is a sensitive but not specific test for ankylosing spondylitis [2]. The sensitivity of the modified Schober index is 25%, while the specificity is 95% [12]. 

The range of motion of the hips is also examined to include this as a possible pain-generating factor.

#### 3.2.4. Neurological Examination

The neurological examination of the lumbar spine consists of a sensory examination and a motor examination. Furthermore, we test the patient’s reflexes and look for any signs of pyramidal tract dysfunction [2,9,15].

##### Sensory Examination

**Figure 1:**
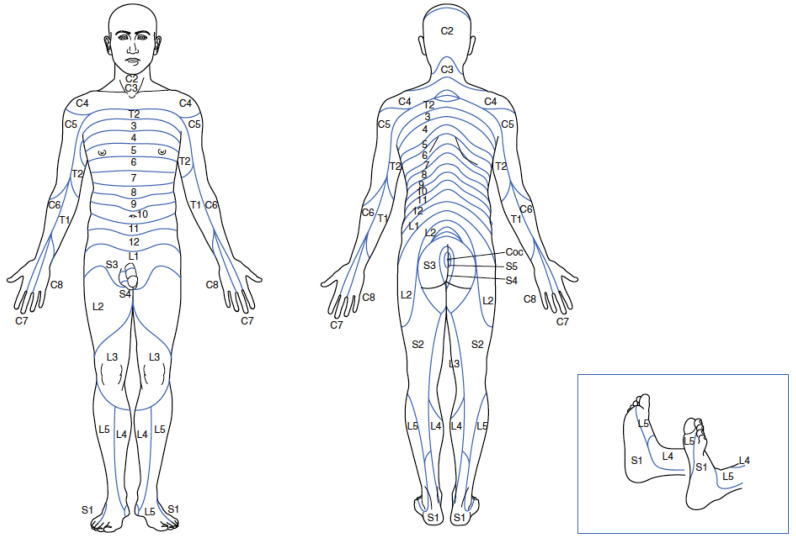
Dermatome map. Note: figure from Braddom et al. [12].

When evaluating the sensory system, knowing the dermatomes is fundamental. A dermatome is a skin area supplied by one spinal nerve root. Both superficial (touch, pain, and temperature) and deep sensations (perception of position/vibration) are tested. Figure 1 shows the dermatome map for each spinal nerve root [2,5,7,15]. 

##### Motor Examination

The motor examination consists of testing the strength of the lower limb muscles, coordination, and tone. It is important to note that most muscles in the limb receive innervation from more than one spinal nerve root, so there is an important overlap in myotome distribution [2,7,15].

##### Reflexes

Another part of the neurological examination is testing of the reflexes. The reflexes can be divided into pathological and muscle stretch reflexes. The pathological reflex is called the plantar reflex of the Babinski sign. Here, the physician applies a stimulus to the sole from the lateral border up to the ball of the foot. This test is positive when there is an extension of the big toe and abduction of the other toes. The muscle stretch reflexes of the lower limb exist in the patellar and Achilles reflexes. Hyporeflexia of these reflexes can mean a radicular pattern, respectively, of L2–L4 and S1–S2 pathology [2,15]. See Table 3 for an overview. 

There is a grading system available for the reflexes. The score varies from 0 to 4. A score of 0 means no response of the reflexes. Score 1 is assigned when there is hyporeflexia, score 2 is considered normal, score 3 is when there is hyperreflexia, and lastly, score 4 means hyperreflexia with clonus [2,15]. 

The sensitivity and specificity of the neurological examination were studied by Nezari et al. [20]. In this study, the sensory, motoric, and reflex examination were compared to the results of surgery, radiology (MR, CT, and myelography), and radiological findings. The results are presented in Table 4. In general, tests for sensory deficits exhibit higher specificity with imaging, particularly when conducted at the segmental level, compared to surgery. Motor deficits are better detected through imaging at the segmental level than through surgery, although the sensitivity remains relatively low. For reflex deficits, surgery has slightly higher specificity; however, sensitivity remains low for both surgery and imaging. This summary indicates that imaging, especially at the segmental level, often has higher specificity, while the sensitivity for all types of deficits remains quite low, suggesting a significant likelihood of both false negatives and false positives depending on the chosen test [20].

It is important to look for the “red flags” in the history of the patient, but also in the neurological examination. Two clinical findings call for urgent action. Firstly, pyramidal signs of the spine indicate that the pyramidal tract is affected. The symptoms vary but can consist of hyperreflexia, weakness, spasticity, and the Babinski sign. Secondly, symptoms of urinary retention, urinary and/or fecal incontinence, saddle anesthesia, weakness or paralysis of the lower extremities, pain in the back/legs, and sexual dysfunction can point to a cauda equina syndrome. This occurs when there is significant compression on the lumbar and sacral nerve roots [2,7,13].

#### 3.2.5. Specific Tests of the Lumbar Spine

We also use several specific tests for the lumbar spine, which will be explained in this section, along with their relevance. We did not find sensitivity or specificity data for every test when reviewing the literature. However, in this section, we aim to provide an overview of the sensitivity and specificity of the tests for which we obtained available information.

##### Straight Leg Raising

When performing the Straight Leg Raising (SLR) test, the patient’s leg is raised when lying in a supine position with the knee extended until the patient begins to feel pain radiating to the limbs. The type and distribution of the pain as well as the angle of elevation are recorded. The test is positive when the angle is between 30 and 70 degrees and pain is reproduced down the posterior thigh below the knee. A positive test can indicate neural tension of the L5 and S1 roots [2,5,15]. 

The Straight Leg Raising (SLR) test demonstrates high sensitivity, with values consistently above 0.73, reaching up to 0.98 in some cases. This high sensitivity indicates that the SLR test is effective for identifying true-positive cases, making it a reliable choice for diagnosing conditions related to neural tension. However, its specificity ranges from 0.10 to 1, which suggests a substantial rate of false positives. Therefore, while the SLR is a robust tool for ruling in neural tension, it may not be as effective for ruling it out [2,21,22,23,24].

##### Crossed Straight Leg Raise

The Crossed Straight Leg Raise test is the same as the straight leg test, but the contralateral leg is raised. The test is positive when the patient starts to feel pain down the posterior thigh below the knee in the ipsilateral leg when the angle is between 30 and 70 degrees [2,5]. 

In contrast, the Crossed Straight Leg Raise (Crossed SLR) test exhibits lower sensitivity, with values between 0.23 and 0.43, indicating limited ability to detect true-positive cases. Nevertheless, it achieves high specificity, ranging from 0.88 to 0.98, which means it excels at correctly identifying true negatives and minimizing false positives. This test is particularly useful for confirming the absence of neural tension, providing a reliable diagnostic tool when specificity is paramount [2,5,21,23].

##### Bragard’s Test

This is an addition to the Straight Leg Raising test and only needs to be performed when the Straight Leg Raising test is positive. In this test, the Straight Leg Raising test is performed, but the leg is lowered just below the pain threshold. At that moment, the ankle is pulled in dorsiflexion. The test is positive when a recognizable pain is produced [2].

##### Slump Test

The patient is seated with legs together on the table. First, the patient performs a thoracal and lumbar flexion as far as possible. Secondly, the patient is asked to flex the head. Lastly, the patient is asked to extend the knee, and dorsiflexion of the ankle is added by the examiner. The test is positive when neurological symptoms can be reproduced [2,14].

##### Femoral Nerve Stretch 

The patient lies in a prone position. The knee is flexed, and the examiner produces an extension in the hip. The test is positive when pain is reproduced in the anterior aspect of the thigh and/or back and is suggestive of lumbar radiculopathy involving the L2, L3, and L4 nerve roots [2,15]. 

The Femoral Nerve Stretch test shows no values of specificity, indicating inconsistency in confirming the diagnosis of lumbar radiculopathy involving the L2, L3, and L4 nerve roots with a positive test result. Despite this, its specificity is relatively high, at 0.84 to 0.95 [12].

##### Kemp’s Test

Kemp’s test is a specific test to reproduce the pain originating from the lumbar facet joints by performing a combined extension/rotation maneuver. The test is positive when the patient’s pain is produced on the ipsilateral side of rotation [2,11]. 

Kemp’s test presents variable sensitivity values (0.23 to 1), indicating poor performance in identifying true-positive cases. Its specificity, ranging from 0.116 to 0.673, is also moderate, suggesting limited utility in clinical practice for this test, as it neither reliably confirms nor excludes the presence of a condition [25].

##### Prone Instability Test 

This test investigates possible lumbar instability. The patient lies in the prone position on the examination table with their legs over the edge so their feet can rest on the floor. First, the examiner applies posterior-to-anterior pressure to individual levels of the lumbar spine when the patient is relaxed. Secondly, the patient is asked to contract the trunk musculature by lifting their legs from the floor. At this position, the posterior-to-anterior pressure is repeated. The test is positive when pain is experienced in a relaxed position and the pain diminishes or disappears with contraction of the trunk muscles. 

The Prone Instability test offers a balanced diagnostic performance, with a moderate sensitivity of 0.72 and specificity of 0.58. This suggests that the test is reasonably effective for both identifying true-positive cases and minimizing false negatives, making it a viable option in situations where a balanced approach to sensitivity and specificity is needed [26].

##### Summary of Specificity and Sensitivity

In summary, the SLR test is highly sensitive and useful for detecting the presence of a condition, while the Crossed SLR test, with its high specificity, is more suitable for ruling out conditions. The Femoral Nerve Stretch test, despite its variability in sensitivity, maintains good specificity, suggesting its potential utility in confirming diagnoses. Kemp’s test shows limited diagnostic value due to its moderate specificity and sensitivity. The Prone Instability test, with moderate sensitivity and specificity, provides a balanced approach for diagnostic purposes.

These findings highlight the importance of selecting appropriate diagnostic tests based on the clinical context and the need to balance sensitivity and specificity to achieve accurate diagnoses, thereby optimizing patient outcomes. Further research and standardization of test protocols may enhance the diagnostic utility of these tests, particularly for those with currently variable sensitivity. An overview of the sensitivity and specificity rates for each test, with the sources added, can be found in Table 5.

### 3.3. Specific Test of the Hips

As mentioned before, it is important to also perform a full examination of the hips. This examination consists of observation, testing range of motion, and specific testing. Here, we will not fully explain all the specific tests of the hips, as this falls outside of the scope. However, if any of these tests seem to be positive, it is important to notice that the pain refers to the hips.

### 3.4. Specific Testing of the Sacroiliac Joint

One of the most common causes of low back pain (30%) comes from the sacroiliac joint [27]. The specific testing of the sacroiliac joint consists of the Patrick test and the provocation tests [2,27]. In the following sections, we explain how to perform these tests.

#### 3.4.1. Patrick Test (FABERE)

This test consists of a combined flexion, abduction, external rotation, and extension (FABERE) movement. The patient lies in a supine position. The contralateral leg lies in an extended position, while the ankle of the tested leg is placed above the patella of the opposite leg (sign ‘4’). The knee is depressed, while the opposite anterior superior iliac spine (ASIS) is pressed. The test is positive if the patient complains of pain before the knee reaches the level of the opposite site [2,27,28]. 

#### 3.4.2. Provocation Tests

The provocation tests are distraction, compression, thigh thrust, Gaenslen’s test, and the shear test. These tests aim to elicit the pain coming from the sacroiliac joints. 

##### Distraction

The patient is lying in a supine position and the examiner palpates the ASIS and applies a dorsal compression [2,27,28].

##### Compression

The patient is lying in a side bend position. The examiner applies a compression from the ventral iliac crest to the table [2,27,28].

##### Thigh Trust

The patient is lying in a supine position with the unilateral hip and knee flexed at 90 degrees. The examiner places one hand under the sacrum and, with the other hand, (s)he applies a pressure toward the table [2,27,28]. 

##### Gaenslen’s Test

The patient is lying in a supine position at the edge of the table, and the leg to be examined is off the table. The contralateral leg is flexed in the knee and hip and the patient is asked to hold the leg with his/her hands. Then, the examiner gradually moves the ipsilateral leg into hyperextension of the hip. When the test triggers pain, it is positive [2,27,28].

##### Shear Test

The patient lies in a prone position while the examiner applies a ventral compression on the sacrum [2,27,28].

#### 3.4.3. Specificity and Sensitivity

The evidence of the sacroiliac tests is less investigated. A study by Telli et al. from 2008 studied the reliability and validity of the sacroiliac tests. The results of this study showed that the diagnosis of sacroiliac dysfunction can be made when three or more provocation tests are positive. The highest sensitivity was found in the FABERE test (91.4%), and the lowest probability was in the Gaenslen test [28].

## 4. Treatment

In this section, the conservative treatment of CNSLBP is discussed.

### 4.1. Non-Pharmacological Treatment

It is important to discuss all the treatment options with the patient and to choose the treatment the patient prefers, because this leads to better outcomes [5,29].

#### 4.1.1. Reassurance and Patient Education

It is important to explain to the patient correctly and understandably. When the diagnosis is that of a CNSLBP, the patient must be reassured that there is no serious underlying pathology, and that the prognosis is good. It can be helpful to explain the natural course of their pain, to advise on how to manage their pain, and to tell them to return to normal activities as soon as possible [5,6,9,30]. The grade of health literacy and psychosocial factors must be considered [31]. The result of the treatment depends greatly on the expectations of the patient [32].

When reviewing the evidence, there is strong evidence that in subacute LBP, an individual education session of 2.5 h is effective for both short-term and long-term return-to-work, while in CNSLBP, individual education is less effective [33]. 

#### 4.1.2. Manual Techniques

Manual techniques consist of manipulation, mobilization, and soft tissue techniques. There is no reliable evidence for using these techniques alone, but there is some clinical benefit when combining them with an active treatment [6]. For patients with acute LBP, the results are inconclusive, and for chronic LBP, there is only a limited effect on pain at one month but no significant effect in the long term [5,34,35,36]. 

#### 4.1.3. Massage

In an acute setting, massage can have a good effect on pain in the short term. When LBP is chronic, no or a small effect on long-term function and pain is seen [34,36,37]. 

#### 4.1.4. Acupuncture/Trigger Point Therapy

Positive results of acupuncture and trigger point therapy are reported in acute and chronic LBP, but without evidence. For patients with chronic LBP, pain is statistically lower immediately after the treatment and in the short term only [36,38,39,40,41,42].

#### 4.1.5. Lumbar Supports

There is limited evidence that lumbar support is effective in relieving acute low back pain. There is no evidence that it will prevent the reoccurrence of the pain [2,5,43]. 

#### 4.1.6. Psychological Intervention 

Psychological interventions consist of progressive relaxation, electromyographic feedback, and cognitive behavioral therapy. In the short term, there is a positive result on pain, but not in the long term or on functional status [34,44]. In contrast, the BEST trial showed a long-term benefit [45].

#### 4.1.7. Kinesiotape

The current evidence suggests that kinesiotape can be beneficial for short-term pain reduction and disability improvement in individuals with chronic low back pain. However, its effectiveness compared to other established therapies remains inconclusive, and it does not provide additional benefits when combined with exercise and manual therapy. More high-quality studies are needed to establish the long-term efficacy of kinesiotape in managing chronic low back pain [46,47,48].

#### 4.1.8. Exercise 

Patients with low back pain (LBP) must remain active. Various exercise programs are available, ranging from general exercises (such as yoga, Pilates, aerobic exercises, stretching, strength training, and mind–body exercises), to the McKenzie method, to muscle control exercises [4,9,49,50,51,52]. These can be performed either individually or in groups. Besides that, the back school program is frequently mentioned in the literature [2,53,54,55,56]. This program includes lumbar stabilization exercises, core strengthening, and motor control exercises, though no standardized version exists. Another widely used exercise model is the McKenzie method, which is an individualized exercise program based on clinical observations during assessment [57]. 

Exercise therapy is recommended as a first-line treatment for patients with radicular pain. However, evidence suggests that exercise is only slightly more effective than no treatment in the short term, and long-term benefits are uncertain [10].

The broad nature of exercise makes it challenging to identify a superior type, program, intensity, or duration from the available literature [6]. Our perspective is that exercise should be categorized into strength training and lumbar spine stabilization programs (including motor control exercises).

The effectiveness of exercise on LBP varies depending on the study. Oliveira et al. found some efficacy of exercise in both acute and chronic LBP. Conversely, recent Cochrane reviews indicate a distinction between acute and chronic LBP. For acute LBP, there is no clinically relevant short-term effect on pain and function, with uncertain evidence [58]. In chronic LBP, exercise appears to be more effective for pain than no treatment, showing a clinically relevant reduction in pain and disability, but not in function [49,59]. In contrast, a meta-analysis by Owen et al. showed a positive effect on pain and function of motor control exercises (MCE), resistance training, Pilates, yoga, aerobic, multimodal, and ‘other’ exercise training [4]. Motor control exercises (MCE) are not significantly more effective than other exercises, with evidence ranging from low to high quality. Compared to minimal intervention, MCE shows low- to moderate-quality evidence for short-term pain improvement and a moderate effect size for intermediate and long-term follow-up [60]. A recent meta-analysis by Fernández et al. investigated the superiority of exercise types, regarding pain and disability. All types of exercise therapy were effective, except for stretching exercises for pain, and for disability, the McKenzie method did not seem to be effective. Pilates was the most effective intervention regarding pain, followed by mind–body and core exercises [49,50,51]. Pilates was also the most effective treatment concerning disability, followed by strength and core stability training [4,49,50]. There is strong evidence that yoga improves pain in the short and long term [61]. 

Based on the characteristics of exercise programming, it appears that the following factors may be more effective for managing pain and disability compared to other exercise interventions: (1) engaging in at least 1 to 2 sessions per week of Pilates or strength training, (2) limiting session duration to less than 60 min, focusing on core-based, strength, or mind–body exercises, and (3) implementing training programs that span 3 to 9 weeks, emphasizing Pilates and core-based exercises [49]. 

Regarding the back school program, three Cochrane reviews were analyzed: one focused on (sub)acute LBP and the other two on chronic LBP. For (sub)acute LBP, the effectiveness of back schools is uncertain due to very low-quality evidence [53]. Some studies indicate that back schools reduce pain and improve function in the short and intermediate term for chronic LBP [2,54,55]. However, a 2017 study by Parreira et al. found low- to very-low-quality evidence, making it uncertain whether back schools are effective for chronic LBP [56]. 

#### 4.1.9. Multidisciplinary Biopsychosocial Rehabilitation (MBR)

Multidisciplinary biopsychosocial rehabilitation (MBR) combines an exercise program with psychological, social, educational, or ergonomic treatments, aimed at functional restoration. Various studies suggest benefits from this approach, although the costs are often deemed non-beneficial [6,62].

At our center, we use the David Spine Concept (DSC) for multidisciplinary rehabilitation. The DSC involves a quantitative assessment of lumbar muscle strength and range of motion. A recent study demonstrated significant improvements (*p* < 0.001) in disability, pain, and functional status with DSC [63]. 

Our program for patients with chronic lumbar pain includes 28 sessions, conducted twice weekly for 2 h each. The program focuses on strengthening the muscles around the spinal column, which provides better support for pain-provoking structures. Additionally, the program includes warm-up exercises, floor exercises, an extensive back school and ergonomics program, and sessions with a psychologist and occupational therapist.

Several studies indicate that MBR reduces pain and disability in the short and long term for subacute and chronic LBP [2,34,62,64,65]. For subacute LBP, the evidence for pain reduction is moderate, while for disability, it is low. A 2017 study by Marin et al. reported low evidence for improved work return and fewer sick days. When compared to other treatments, MBR showed no significant differences [65]. For chronic LBP, MBR offers moderate- to low-quality evidence of being more effective than usual care in reducing pain and disability, with no differences in long-term work outcomes [64].

## 5. Some Specific Pathologies

The goal of the history and clinical examination is to identify the structures responsible for low back pain (LBP). If the causative structures can be determined, specific treatments can be recommended. This section outlines the findings in the history and clinical examination of different sources of pain, followed by recommended treatment options based on our perspective. 

### 5.1. Anterior Element Pain 

The anterior vertebral column includes the vertebral body and the intervertebral discs, both of which can cause discogenic pain and are stressed by similar positions. Patients with pain originating from the anterior elements often report mechanical pain in the midline of the back, which worsens with forward bending or lifting objects. They may also experience pain when riding a bike over potholes. Clinical examination typically reveals a positive Gower sign and Prone Instability test, while the neurological examination remains normal. Degenerative vertebral alterations can be identified on magnetic resonance imaging (MRI) as Modic changes. These changes are classified into three types. Type I Modic changes are characterized by bone marrow edema and inflammation, and they are strongly correlated with the presence of lower back pain [13]. The recommended treatment includes adequate pain medication and initiating physical therapy. In chronic cases, referral to multidisciplinary rehabilitation is advised. 

### 5.2. Unilateral Facet Joint Pain 

Lumbar facet joint pain is characterized by mechanical pain that increases with lumbar spine extension. Pain may radiate to the buttocks, lateral thighs, or groin, but typically not beyond the knee. Clinical examination usually shows pain upon palpation of the facet joints and during extension, lateral bending, and rotation. A positive Kemp’s test is often observed, while the neurological examination remains normal [11]. The recommended treatment is McKenzie therapy. 

### 5.3. Sacroiliac Dysfunction 

Pain originating from the sacroiliac joint is typically located in the gluteal region and may radiate to the groin, thigh, or occasionally below the knee. Patients often report worsening pain with prolonged sitting. Clinical examination reveals pain upon palpation of the sacroiliac joint, with negative specific tests for the lumbar spine and hip, but positive specific tests for the sacroiliac joint. The neurological examination is normal. Recommended treatment includes exercises focusing on pelvic stabilization and core stability [27,28]. 

### 5.4. Hip Pathology 

Pain originating from the hips typically presents with complaints such as pain while putting on shoes or climbing stairs. The lumbar spine, sacroiliac joint, and neurological examinations are normal. However, hip examination may reveal a restricted range of motion and positive specific tests. Treatment of hip pathologies is outside the scope of this discussion but is an important differential diagnosis to consider. 

### 5.5. Other Pathologies 

When history or clinical examination suggest urgent or alternative pathologies, further investigations are necessary to rule out conditions such as spondylolysis, fractures, infections, or cancer. It is important to be vigilant regarding fractures following trauma and throbbing pain during clinical examination. Spondylolysis should be considered in young athletes engaged in rotational sports. Non-mechanical pain at rest and night pain may indicate infection. If the neurological examination suggests radiculopathy, further investigations with MRI and ENMG are warranted to adjust the treatment accordingly [2,7,13]. 

## 6. Conclusions

Chronic non-specific low back pain is a common condition, with a prevalence of 85%. This review aims to provide an evidence-based approach to low back pain and its conservative treatment. Initially, it is essential to exclude “red flags” during patient history. Identifying whether the pain is specific/non-specific or radicular/radiculopathy is crucial. A standardized protocol for history taking, including duration, onset, irradiation, aggravating factors, and nocturnal pain, is beneficial. It is also important to identify patients at high risk for chronic pain by considering psychological, psychiatric, contextual, and work-related factors, categorized as yellow, orange, black, and blue flags. Questionnaires, such as the OLBPDQ, Tampa Scale, and Zung Self-Rating Depression Scale, may be useful. 

In our center, we use a standardized protocol for the physical examination of the lumbar spine, hips, and sacroiliac joint, which includes observation, palpation, range of motion, neurological examination, and specific tests. The findings from these examinations must be integrated. In the neurological examination, it is crucial to identify urgent cases, such as pyramidal signs and cauda equina syndrome. Muscle weakness, sensory deficits, and reflex deficits related to specific dermatomes/myotomes should be noted. For radiculopathy involving the L5 and S1 roots, the Straight Leg Raising test has high sensitivity, while the Crossed Straight Leg Raising test, Braggard’s sign, and bowstring sign should also be assessed. For lumbar radiculopathy involving the L2, L3, and L4 nerve roots, the Femoral Nerve Stretch test has high specificity and sensitivity. Kemp’s test, which assesses facet joint pain, and the Prone Instability test, which evaluates lumbar spine instability, are also important. 

For CNSLBP, conservative management is recommended. Initial treatment includes reassurance and patient education, explaining the condition clearly and understandably. While manipulation, massage, acupuncture, and lumbar supports can be added, evidence for their efficacy is lacking. Our center focuses on exercise as a treatment modality, despite the lack of a standardized protocol. We advocate combining exercise programs with psychological, social, educational, and ergonomic treatments within a multidisciplinary biopsychosocial rehabilitation program. Although evidence for the effectiveness of multidisciplinary treatment is limited, we have observed positive outcomes with the David Spine Concept (DSC) in our center. Both subjective and objective improvements in mobility and strength are noted, and our data indicate that patients with chronic low back pain have a higher likelihood of returning to work after multidisciplinary rehabilitation. Therefore, we strongly recommend a multidisciplinary approach to CNSLBP. 

## Figures and Tables

**Table 1:** Red flags. *Note: Data taken from table in ‘Klinische Richtlijn rond lage rugpijn en radiculaire pijn’* [7]. Blue arrow: If a red flag symptom is present, please refer to the action plan.

**Urgent**		
**Red flag**		**Action**
**Neurological** - Widespread neurological signs: pyramidal signs, coordination problems, and motoric weakness - Progressive neurological symptoms - Saddle anesthesia, hypoesthesia, urinary retention, fecal and urinary incontinence, and sexual dysfunction - Motoric deficit (MRC <= 3/5) in less than 48 h	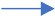	Refer patient to emergency care
**Traumatic fractures** - Severe low back pain after significant/high-energy trauma OR - Back pain after trauma with ankylosing spondylitis	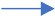	Refer patient to emergency care
**Vascular issues** - Vascular signs (cold foot and decreased peripheral arterial pulsation) that may indicate a ruptured aortic aneurysm if accompanied by low back pain or even shock	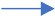	Refer patient to emergency care Ultrasound and vascular surgery consultation
**Semi-urgent (in 48 h)**		
**Red flag**		**Action**
**Pathological fracture** Low back pain after minor trauma or even without a notion of trauma with: - History/risk of osteoporosis - Chronic corticoid use - Pain in the chest - Higher age - Unexplained weight loss and fatigue - History of cancer	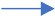	X-ray (or CT) and consultation with a spine specialist
**Infection** - Objective signs (e.g., night sweats, fever, and chills) - Intravenous drug use - Immunodeficient patient - Unexplained weight loss - Known previous or concurrent systemic infection or risk of infection - Recent surgical procedure - Urinary or skin infection	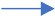	- MRI of lumbar spine - Blood analysis (e.g., leucocyte count, CRP, and sedimentation) - Consultation with a spine specialist and/or consultation with internal medicine
**Less urgent**		
**Red flag**		**Action**
**Tumor** - Recent complaints of back pain at age < 18 or >55 - History of cancer - Unexplained weight loss and fatigueSevere night pain	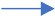	- MRI of lower back - Oncology consultation - Spine specialist consultation
**Inflammatory condition** - Constant progressive non-mechanical pain - Back pain improves with exercise, but not with rest - Severe pain at night - Morning stiffness > 30 min or nighttime awakenings in younger patients	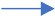	Consultation—rheumatology
**Miscellaneous** - Increasing postoperative pain - Unbearable and treatment-resistant low back pain (>6 weeks) - Unilateral pyramidal signs	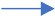	- MRI - Consultation with a spine specialist, i.e., a specialist in physical medicine and rehabilitation, an orthopedic surgeon or neurosurgeon, or an anesthetist-algologist

**Table 2:** Yellow, orange, black, and blue flags [7].

Flag	Explanation
**Yellow flag** [2,6,8,16]	Beliefs and perceptions Emotional responses to pain
**Orange flags**	Psychiatric symptoms (depression or personality disorder)
**Black flags**	System or contextual matters (e.g., profession, family, etc.)
**Blue flags**	Perception of the relationship between work and health

**Table 3:** Overview of pathological and muscle stretch reflexes.

				**When Pathological?**
**Pathological reflex**	Plantar reflex Babinski sign	Stimulus is applied to the sole from the lateral border up to and across the ball of the foot		Extension of the big toe and abduction of the other toes
Muscle stretch reflex (lower limb)	Assessed by tapping over the muscle tendon with a reflex hammer			Hyporeflexia Hyperreflexia +− Clonus
	Patella	Femoral nerve	L2–L4	
	Achilles	Tibial nerve	S1–S2	

**Table 4:** Sensitivity and specificity of sensory, motor, and reflex deficits. Note: table is taken from Nezari et al. [20].

Index Test	Gold Standard	Sensitivity (95% CI)	Specificity (95% CI)
Sensory deficit	Surgery	0.40 (0.38, 0.43)	0.59 (0.51, 0.67)
	Imaging	0.32 (0.28, 0.37)	0.72 (0.67, 0.77)
	Imaging by level	0.35 (0.33, 0.38)	0.64 (0.61, 0.66)
Motor deficit	Surgery	0.22 (0.21, 0.23)	0.79 (0.77, 0.80)
	Imaging by level	0.40 (0.37, 0.42)	0.62 (0.60, 0.64)
Reflex deficits	Surgery	0.29 (0.28, 0.30)	0.78 (0.76, 0.80)
	Imaging by level	0.25 (0.22, 0.28)	0.75 (0.73, 0.78)

**Table 5:** Summary of sensitivity and specificity of each test.

Test	Sensitivity	Specificity	Source
Straight Leg Raise	0.85 0.91 0.73–0.98 0.92	0.28 0.52 0.26 0.11–0.61 0.10–1	[21] [22] [23] [2] [24]
Crossed SLR	0.28 0.29 0.23–0.43	0.90 0.88 0.88–0.98	[21] [23] [2]
Femoral Nerve Stretch	0.84–0.95		[12]
Kemp’s test	0.23–1	0.116–0.673	[25]
Prone Instability test	0.72	0.58	[26]

## References

[1] Sciensano Welke Impact Hebben de 37 Voornaamste Ziekten op de Gezondheid van de Belgen?. https://www.sciensano.be/nl/pershoek/welke-impact-hebben-de-37-voornaamste-ziekten-op-de-gezondheid-van-de-belgen.

[2] Cifu D., Eapen B.C., Johns J.S., Kowalske K., Lew H.L., Miller M.A., Worspwocz G. (2021). Braddom’s Physical Medicine and Rehabilitation.

[3] World Health Organization Low Back Pain. https://www.who.int/news-room/fact-sheets/detail/low-back-pain.

[4] Owen P.J., Miller C.T., Mundell N.L., Verswijveren S., Tagliaferri S.D., Brisby H., Bowe S.J., Belavy D.L. (2020). Which specific modes of exercise training are most effective for treating low back pain? Network meta-analysis. Br. J. Sports Med..

[5] Chou R., Qaseem A., Snow V., Casey D., Cross J.T., Shekelle P., Owens D.K. (2007). Diagnosis and Treatment of Low Back Pain: A Joint Clinical Practice Guideline from the American College of Physicians and the American Pain Society. Ann. Intern. Med..

[6] Oliveira C.B., Maher C.G., Pinto R.Z., Traeger A.C., Lin C.-W.C., Chenot J.-F., van Tulder M., Koes B.W. (2018). Clinical practice guidelines for the management of non-specific low back pain in primary care: An updated overview. Eur. Spine J..

[7] Van Wambeke P., Desomer A., Ailliet L., Berquin A., Demoulin C., Depreitere B., Dewachter J., Dolphens M., Forget P., Fraselle V. (2017). Klinische Richtlijn Rond Lage Rugpijn en Radiculaire Pijn—Samenvatting.

[8] Rubinstein S.M., van Tulder M. (2008). A best-evidence review of diagnostic procedures for neck and low-back pain. Best Pract. Res. Clin. Rheumatol..

[9] Maher C., Underwood M., Buchbinder R. (2017). Non-specific low back pain. Lancet.

[10] Peene L., Cohen S.P., Kallewaard J.W., Wolff A., Huygen F., Gaag A.V., Monique S., Vissers K., Gilligan C., Van Zundert J. (2024). 1. Lumbosacral radicular pain. Pain Pract..

[11] Heuvel S., Cohen S., Boxem K., Ares J., Kallewaard J., Fipp J. (2024). 3. Pain originating from the lumbar facet joints. Pain Pract..

[12] Cifu D.X., Eapen B.C. (2011). Braddom’s Physical Medicine and Rehabilitation.

[13] Tuchman A. (2017). Surgery of the Spine and Spinal Cord, Erik Van de Kelft, editor (Ed.), Springer International Publishing (2016), p. 761 pages, ISBN 978-3-319-27611-3. World Neurosurg..

[14] Brukner P., Clarsen B., Cook J., Cools A., Crossley K., Hutchinson M., McCrory P., Bahr R., Khan K. (2017). Brukner & Khan’s Clinical Sports Medicine: Injuries, Volume 1, 5e.

[15] Hemmer C.R. (2021). Evaluation and Treatment of Low Back Pain in Adult Patients. Orthop. Nurs..

[16] Nicholas M.K., Linton S.J., Watson P.J., Main C.J. (2011). Early identification and management of psychological risk factors (“yellow flags”) in patients with low back pain: A reappraisal. Phys. Ther..

[17] Harlick J.C., Milosavljevic S., Milburn P.D. (2007). Palpation identification of spinous processes in the lumbar spine. Man. Ther..

[18] Nolet P.S., Yu H., Côté P., Meyer A.L., Kristman V.L., Sutton D., Murnaghan K., Lemeunier N. (2021). Reliability and validity of manual palpation for the assessment of patients with low back pain: A systematic and critical review. Chiropract. Man. Ther..

[19] Wong C.K., Johnson E.K. (2012). A Narrative Review of Evidence-Based Recommendations for the Physical Examination of the Lumbar Spine, Sacroiliac and Hip Joint Complex. Musculoskelet. Care.

[20] Al Nezari N.H., Schneiders A.G., Hendrick P.A. (2013). Neurological examination of the peripheral nervous system to diagnose lumbar spinal disc herniation with suspected radiculopathy: A systematic review and meta-analysis. Spine J. Off. J. N. Am. Spine Soc..

[21] Deyo R.A., Mirza S.K. (2016). Herniated Lumbar Intervertebral Disk. N. Engl. J. Med..

[22] Vroomen P.C., de Krom M.C., Knottnerus J.A. (1999). Diagnostic value of history and physical examination in patients suspected of sciatica due to disc herniation: A systematic review. J. Neurol..

[23] Devillé W.L., van der Windt D.A., Dzaferagić A., Bezemer P.D., Bouter L.M. (2000). The test of Lasègue: Systematic review of the accuracy in diagnosing herniated discs. Spine.

[24] van der Windt D., Simons E., Riphagen I.I., Ammendolia C., Verhagen A.P., Laslett M., Devillé W., Deyo R.A., Bouter L.M., de Vet H.C.W. (2010). Physical examination for lumbar radiculopathy due to disc herniation in patients with low-back pain. Cochrane Database Syst. Rev..

[25] Stuber K., Lerede C., Kristmanson K., Sajko S., Bruno P. (2014). The diagnostic accuracy of the Kemp’s test: A systematic review. J. Can. Chiropract. Assoc..

[26] Hicks G.E., Fritz J.M., Delitto A., McGill S.M. (2005). Preliminary Development of a Clinical Prediction Rule for Determining which Patients with Low Back Pain Will Respond to a Stabilization Exercise Program. Arch. Phys. Med. Rehabil..

[27] Buchanan P., Vodapally S., Lee D.W., Hagedorn J.M., Bovinet C., Strand N., Sayed D., Deer T. (2021). Successful Diagnosis of Sacroiliac Joint Dysfunction. J. Pain Res..

[28] Telli H., Telli S., Topal M. (2018). The Validity and Reliability of Provocation Tests in the Diagnosis of Sacroiliac Joint Dysfunction. Pain Physician.

[29] Kalauokalani D., Cherkin D.C., Sherman K.J., Koepsell T.D., Deyo R.A. (2001). Lessons from a trial of acupuncture and massage for low back pain: Patient expectations and treatment effects. Spine.

[30] Waddell G., Feder G., Lewis M. (1997). Systematic reviews of bed rest and advice to stay active for acute low back pain. Br. J. Gen. Pract..

[31] Glassman S.D., Carreon L.Y., Brown M.E., Jones J.S., Edward J., Li J., Williams M.V. (2019). The impact of health literacy on health status and resource utilization in lumbar degenerative disease. Spine J..

[32] Hayden J.A., Wilson M.N., Riley R.D., Iles R., Pincus T., Ogilvie R. (2019). Individual recovery expectations and prognosis of outcomes in non-specific low back pain: Prognostic factor review. Cochrane Database Syst. Rev..

[33] Engers A.J., Jellema P., Wensing M., van der Windt D., Grol R., van Tulder M.W. (2008). Individual patient education for low back pain. Cochrane Database Syst. Rev..

[34] Chou R., Deyo R., Friedly J., Skelly A., Hashimoto R., Weimer M., Fu R., Dana T., Kraegel P., Griffin J. (2017). Nonpharmacologic Therapies for Low Back Pain: A Systematic Review for an American College of Physicians Clinical Practice Guideline. Ann. Intern. Med..

[35] Rubinstein S.M., van Middelkoop M., Assendelft W.J.J., de Boer M.R., van Tulder M.W. (2011). Spinal Manipulative Therapy for Chronic Low-Back Pain: An Update of a Cochrane Review. Spine.

[36] Furlan A., Yazdi F., Tsertsvadze A., Gross A., Tulder M., Santaguida P., Gagnier J., Ammendolia C., Dryden T., Doucette S. (2012). A Systematic Review and Meta-Analysis of Efficacy, Cost-Effectiveness, and Safety of Selected Complementary and Alternative Medicine for Neck and Low-Back Pain. Evid.-Based Complement. Altern. Med..

[37] Furlan A.D., Giraldo M., Baskwill A., Irvin E., Imamura M. (2015). Massage for low-back pain. Cochrane Database Syst. Rev..

[38] Birch S., Hesselink J.K., Jonkman F.A., Hekker T.A., Bos A. (2004). Clinical research on acupuncture. Part 1. What have reviews of the efficacy and safety of acupuncture told us so far?. J. Altern. Complement. Med..

[39] Lee J.H., Choi T.Y., Lee M.S., Lee H., Shin B.C., Lee H. (2013). Acupuncture for acute low back pain: A systematic review. Clin. J. Pain.

[40] Lam M., Galvin R., Curry P. (2013). Effectiveness of acupuncture for nonspecific chronic low back pain: A systematic review and meta-analysis. Spine.

[41] Furlan A.D., van Tulder M., Cherkin D., Tsukayama H., Lao L., Koes B., Berman B. (2005). Acupuncture and dry-needling for low back pain: An updated systematic review within the framework of the cochrane collaboration. Spine.

[42] Manheimer E., White A., Berman B., Forys K., Ernst E. (2005). Meta-Analysis: Acupuncture for Low Back Pain. Ann. Intern. Med..

[43] Jellema P., van Tulder M.W., van Poppel M.N., Nachemson A.L., Bouter L.M. (2001). Lumbar supports for prevention and treatment of low back pain: A systematic review within the framework of the Cochrane Back Review Group. Spine.

[44] Henschke N., Ostelo R., van Tulder M.W., Vlaeyen J.W.S., Morley S., Assendelft W.J.J., Main C.J. (2010). Behavioural treatment for chronic low-back pain. Cochrane Database Syst. Rev..

[45] Lamb S.E., Mistry D., Lall R., Hansen Z., Evans D., Withers E.J., Underwood M.R., On behalf of the Back Skills Training Trial Group (2012). Group cognitive behavioural interventions for low back pain in primary care: Extended follow-up of the Back Skills Training Trial (ISRCTN54717854). Pain.

[46] Pan L., Li Y., Gao L., Sun Y., Li M., Zhang X., Wang Y., Shi B. (2023). Effects of Kinesio Taping for Chronic Nonspecific Low Back Pain: A Systematic Review and Meta-analysis. Altern. Ther. Health Med..

[47] Lin S., Zhu B., Huang G., Wang C., Zeng Q., Zhang S. (2020). Short-Term Effect of Kinesiotaping on Chronic Nonspecific Low Back Pain and Disability: A Meta-Analysis of Randomized Controlled Trials. Phys. Ther..

[48] Sheng Y., Duan Z., Qu Q., Chen W., Yu B. (2019). Kinesio taping in treatment of chronic non-specific low back pain: A systematic review and meta-analysis. J. Rehabil. Med..

[49] Fernández-Rodríguez R., Álvarez-Bueno C., Cavero-Redondo I., Torres-Costoso A., Pozuelo-Carrascosa D.P., Reina-Gutiérrez S., Pascual-Morena C., Martínez-Vizcaíno V. (2022). Best Exercise Options for Reducing Pain and Disability in Adults with Chronic Low Back Pain: Pilates, Strength, Core-Based, and Mind-Body. A Network Meta-analysis. J. Orthop. Sports Phys. Ther..

[50] Yamato T.P., Maher C.G., Saragiotto B.T., Hancock M.J., Ostelo R., Cabral C.M.N., Menezes Costa L.C., Costa L.O.P. (2015). Pilates for low back pain. Cochrane Database Syst. Rev..

[51] Cherkin D.C., Herman P.M. (2018). Cognitive and Mind-Body Therapies for Chronic Low Back Pain and Neck Pain: Effectiveness and Value. JAMA Intern. Med..

[52] Hilde G., Hagen K.B., Jamtvedt G., Winnem M. (2006). Advice to stay active as a single treatment for low-back pain and sciatica. Cochrane Database Syst. Rev..

[53] Poquet N., Lin C.W.C., Heymans M.W., van Tulder M.W., Esmail R., Koes B.W., Maher C.G. (2016). Back schools for acute and subacute non-specific low-back pain. Cochrane Database Syst. Rev..

[54] Heymans M.W., van Tulder M.W., Esmail R., Bombardier C., Koes B.W. (2004). Back schools for non-specific low-back pain. Cochrane Database Syst. Rev..

[55] Hernandez-Lucas P., Leirós-Rodríguez R., Lopez-Barreiro J., García-Soidán J.L. (2024). The Effects of Back Schools on Non-Specific Back Pain: A Systematic Review and Meta-Analysis. J. Pers. Med..

[56] Parreira P., Heymans M.W., van Tulder M.W., Esmail R., Koes B.W., Poquet N., Lin C.W.C., Maher C.G. (2017). Back Schools for chronic non-specific low back pain. Cochrane Database Syst. Rev..

[57] Almeida M.O., Narciso Garcia A., Menezes Costa L.C., van Tulder M.W., Lin C.W., Machado L.A.C. (2023). The McKenzie method for (sub)acute non-specific low back pain. Cochrane Database Syst. Rev..

[58] W I.J., Oosterhuis T., Hayden J.A., Koes B.W., van Tulder M.W., Rubinstein S.M., de Zoete A. (2023). Exercise therapy for treatment of acute non-specific low back pain. Cochrane Database Syst. Rev..

[59] Hayden J.A., Ellis J., Ogilvie R., Malmivaara A., van Tulder M.W. (2021). Exercise therapy for chronic low back pain. Cochrane Database Syst. Rev..

[60] Saragiotto B.T., Maher C.G., Yamato T.P., Costa L.O.P., Menezes Costa L.C., Ostelo R., Macedo L.G. (2016). Motor control exercise for chronic non-specific low-back pain. Cochrane Database Syst. Rev..

[61] Cramer H., Lauche R., Haller H., Dobos G. (2013). A Systematic Review and Meta-analysis of Yoga for Low Back Pain. Clin. J. Pain.

[62] Guzmán J., Esmail R., Karjalainen K., Malmivaara A., Irvin E., Bombardier C. (2001). Multidisciplinary rehabilitation for chronic low back pain: Systematic review. BMJ.

[63] Langella F., Boido E., Basso S., Bassi C., Biber Z., Vanni D., Damilano M., Berjano P. (2021). Active, Targeted, and Measured Device-Based Therapy for Low Back Pain With the David Spine Concept: Comparison of 2 Treatment Protocols. Top. Geriatr. Rehabil..

[64] Kamper S.J., Apeldoorn A.T., Chiarotto A., Smeets R.J., Ostelo R.W., Guzman J., van Tulder M.W. (2014). Multidisciplinary biopsychosocial rehabilitation for chronic low back pain. Cochrane Database Syst. Rev..

[65] Marin T.J., Van Eerd D., Irvin E., Couban R., Koes B.W., Malmivaara A., van Tulder M.W., Kamper S.J. (2017). Multidisciplinary biopsychosocial rehabilitation for subacute low back pain. Cochrane Database Syst. Rev..

